# Cell-to-Medium Concentration Ratio Overshoot in the Uptake of Statins by Human Hepatocytes in Suspension, but Not in Monolayer: Kinetic Analysis Suggesting a Partial Loss of Functional OATP1Bs

**DOI:** 10.1208/s12248-020-00512-6

**Published:** 2020-10-15

**Authors:** Wooin Lee, Satoshi Koyama, Kiyoe Morita, Aya Kiriake, Ryota Kikuchi, Xiaoyan Chu, Nora Lee, Renato J. Scialis, Hong Shen, Emi Kimoto, Larry Tremaine, Naoki Ishiguro, Ralf Lotz, Kazuya Maeda, Hiroyuki Kusuhara, Yuichi Sugiyama

**Affiliations:** 1 College of Pharmacy and Research Institute of Pharmaceutical Sciences, Seoul National University, 1 Gwanak-ro, Gwanak-gu, Seoul, South Korea; 2 Sugiyama Laboratory, RIKEN Cluster for Science, Technology and Innovation Hub, 1-7-22 Suehiro-cho, Tsurumi, Yokohama, Kanagawa Japan; 3 AbbVie Inc, North Chicago, Illinois USA; 4 Merck & Co., Inc, North Wales, Pennsylvania USA; 5 Daewoong Pharmaceutical Co., Ltd, Seoul, South Korea; 6 Bristol Myers Squibb, Princeton, New Jersey USA; 7 ADME Sciences, Medicine Design, Worldwide Research and Development, Pfizer Inc, Groton, Connecticut USA; 8 Tremaine DMPK Consulting LLC, Merritt Island, Florida USA; 9 Pharmacokinetics and Non-Clinical Safety Department, Nippon Boehringer Ingelheim Co., Ltd, Kobe, Hyogo Japan; 10 Drug Metabolism and Pharmacokinetics, Boehringer Ingelheim Pharma GmbH & Co. KG, Biberach an der Riss, Germany; 11 Laboratory of Molecular Pharmacokinetics, Graduate School of Pharmaceutical Sciences, University of Tokyo, Tokyo, Japan

**Keywords:** Human hepatocytes, Initial uptake clearance, Time course, Statins, Organic anion transporting polypeptide (OATP)

## Abstract

Suspended human hepatocytes (SHH) have long been used in assessing hepatic drug uptake, while plated human hepatocytes in short-term monolayer culture (PHH) have gained use in recent years. This study aimed to cross-evaluate SHH and PHH in measuring the hepatic uptake mediated by organic anion transporting polypeptide 1Bs (OATP1Bs). We compared the time courses of cell-to-medium (C/M) concentration ratios and initial uptake clearance values of the OATP1B substrates (pitavastatin, rosuvastatin, cerivastatin, pravastatin, dehydropravastatin, and SC-62807) between SHH and PHH. For all compounds except cerivastatin, the C/M ratios in SHH displayed an apparent overshoot (an initial increase followed by a decrease) during the 180-min uptake experiment, but not in PHH. Based on the literature evidence suggesting the possible internalization of OATP1Bs in primary hepatocytes, separate experiments measured the drug uptake after varying lengths of pre-incubation in the drug-free medium. The initial uptake clearances of pitavastatin and rosuvastatin declined in SHH beyond an apparent threshold time of 20-min drug-free pre-incubation, but not in PHH. Kinetic modeling quantitatively captured the decline in the active uptake clearance in SHH, and more than half of the active uptake clearances of pitavastatin and rosuvastatin were prone to loss during the 180-min uptake experiment. These results suggested a partial, time-delayed loss of the functional OATP1Bs in SHH upon prolonged incubation. Our results indicate that PHH is more appropriate for experiments where a prolonged incubation is required, such as estimation of unbound hepatocyte-to-medium concentration ratio (K_p,uu_) at the steady-state.

**Electronic supplementary material:**

The online version of this article (10.1208/s12248-020-00512-6) contains supplementary material, which is available to authorized users.

## INTRODUCTION

The liver is a major organ responsible for the elimination of many drugs. The accurate assessment of hepatic clearance is essential in predicting the pharmacokinetic profiles of drug candidates in humans. In the case of anionic molecules, entry to hepatocytes often relies on uptake transporters, which can be the rate-determining step in the overall hepatic drug elimination (1). Thus, it has become a standard practice to assess anionic drug candidates for their uptake clearance at the physiological pH, and to apply the in vitro uptake clearance to the extended clearance concept for prediction of the overall hepatic clearance in humans (1).

During drug discovery and development, the uptake clearance of drug candidates is determined using various in vitro systems and scaled to that in the whole liver. The scaled hepatic clearance is then used along with other organ clearances to predict the total body clearance (2,3). In evaluating hepatic uptake clearance, the available in vitro systems include human primary hepatocytes in suspension (SHH) or short-term monolayer culture (PHH) and more complex three-dimensional cultures (e.g., hepatocyte spheroids, liver-on-a-chip) (4–6). However, complex experimental systems grown in multi-layer or aggregates can hinder access of drug molecules to the hepatocyte surface, making it difficult to quantitatively determine the drug uptake rates per million cells (7). Thus, human primary hepatocytes in the SHH or PHH format continue to be the standard and practical in vitro systems in quantifying the hepatic uptake of drugs.

Previously, we observed that drug uptake (often presented as the cell-to-medium (C/M) concentration ratios) sometimes displays an overshoot phenomenon (an initial increase, followed by a decrease) over time in SHH. For example, the C/M ratios of rosuvastatin (RSV) showed an overshoot during uptake studies using SHH (8). Such an overshoot in the uptake measurements makes it difficult to decide which values (minimum, maximum, or average values) are appropriate in estimating the parameters such as the unbound hepatocyte-to-medium concentration ratio (*K*_p,uu_) at the steady-state (a vital parameter to predict the drug levels available for hepatic metabolism and interactions with intracellular targets (9)). It is thus important to enhance our understanding of the possible sources for the data variability in drug uptake.

The members of the organic anion transporting polypeptide 1B subfamily (OATP1Bs) play an important role in the hepatic uptake of many anionic drugs, including several statins, whose overall hepatic handling is limited by active uptake clearance (1). Like many transmembrane proteins, the expression of OATP1Bs is not static but dynamically regulated by the coordinated processes of de novo synthesis, intracellular processing and trafficking including internalization, and recycling (10,11). For example, protein kinase C (PKC) activation triggers the internalization of OATP1B1 and OATP1B3 thereby decreasing the functional transporter on the cell surface: the functional decline occurred 10–60 min after the addition of a PKC activator to the sandwich-cultured human hepatocytes (SCHH) or HEK293 cells stably expressing OATP1B1 or OATP1B3 (11,12). However, it is currently unknown whether and to what extent the localization and activity of OATP1Bs may change during the period of in vitro uptake studies using human primary hepatocytes in the SHH or PHH format.

The aim of the present study was to cross-evaluate the performance of SHH and PHH in measuring the hepatic uptake of several OATP1B substrates [RSV, pitavastatin (PTV), cerivastatin (CRV), pravastatin (PRV), dehydropravastatin (DHP), and SC-62807 (celecoxib carboxylic acid)]. The time courses of drug uptake (C/M ratios, cellular uptake amounts, and drug concentrations in the medium) were compared between SHH and PHH derived from the same donors. For PTV and RSV, their initial uptake clearances were measured following varying lengths of pre-incubation in drug-free medium. To gain kinetic insights into the observed decline of the uptake clearance, a kinetic model was constructed for the hepatic uptake of PTV and RSV.

## MATERIALS AND METHODS

### Materials and Cells

CRV sodium, PTV calcium, PRV sodium, RSV calcium, and DHP were purchased from Wako Pure Chemicals (Osaka, Japan). SC-62807 (celecoxib carboxylic acid) was purchased from Toronto Research Chemicals (ON, Canada). DHP was synthesized as reported previously (13). Rifamycin SV was purchased from LKT Laboratories (MN, USA). Cryopreserved human hepatocytes of two different lots, each derived from a single donor (lot HH1045, male, 9-year-old, Hispanic; lot HH1052, male, 44-year-old, Caucasian) were purchased from In Vitro ADMET Laboratories (MD, USA). All other reagents and solvents were of analytical grade, purchased from Invitrogen (CA, USA), Sigma-Aldrich (MO, USA), Nacalai Tesque (Japan), or Wako Pure Chemicals (Osaka, Japan).

### Time Course of the Drug Uptake in SHH and PHH (Uptake Study #1)

The time course of the drug uptake (from 0.25 to 180 min) in SHH was evaluated using an oil-filtration method as described previously (8). Cryopreserved human hepatocytes (lots of HH1045, and HH1052) were aliquoted in Krebs–Henseleit buffer (pH 7.4) after counting viable cells via trypan blue staining (10^6^ viable cells suspended/mL). After incubating the suspended hepatocytes at least for 5 min, drug uptake was initiated by adding a cocktail dosing solution containing the six compounds (0.1 μM CRV; 0.1 μM PTV; 0.1 μM RSV; 2 μM PRV; 2 μM DHP; 0.2 μM SC-62807). To minimize risk for mutual interactions among the probe drugs in the cocktail dosing solution, the drug concentrations were chosen to be below their reported K_m_ values for OATP1Bs (Table S1). The drug uptake was terminated by separating cells from the buffer via centrifugation. The drug levels in hepatocytes and medium samples were quantified via liquid chromatography–tandem-mass spectrometry (LC–MS/MS) as described below.

The time course for drug uptake (from 0.25 to 180 min) in PHH was evaluated as follows. After thawing, cryopreserved human hepatocytes were suspended in Universal Primary Cell-Plating Medium (UPCM, In Vitro ADMET Laboratories, MD, USA) and seeded onto 48-well plates at a density of 1.25 × 10^5^ cells/well. Six hours post-plating, the adherent cells were incubated with the cocktail dosing solution (same as described above) in Krebs–Henseleit buffer (pH 7.4) for the pre-defined period (from 0.25 to 180 min). The uptake was terminated by washing the cells three times with ice-cold phosphate-buffered saline. The number of washing steps (i.e., three) was determined based on the results that the drug amount detected in the washing buffer was minimal in the 4th and 5th washing steps during the five sequential washing steps (data not shown). The cells were lysed using a sonication system handling multiple samples simultaneously (Bioruptor, Smith & Nephew, MA, USA), and the drug levels in cell lysates and medium samples were quantified by LC–MS/MS.

Data analysis utilized the following conversion factors: hepatocyte cell volume of 2.28 μL/10^6^ cells (8) (SHH and PHH were assumed to have an equivalent cellular volume), 1 mg total protein/10^6^ cells. The C/M ratios were then calculated as the drug concentrations in cells divided by the drug concentration in the medium.

### Impact of Varying Lengths of Drug-Free Pre-Incubation before the Measurement of the Uptake of PTV and RSV in SHH and PHH (Uptake Study #2)

Our analysis focused on the two compounds PTV and RSV, as they displayed a clear overshoot phenomenon peaking at 60 min in SHH (Uptake study #1; Figs. 2 and 3, S1, and S2). The initial uptake clearances of PTV and RSV were calculated based on the uptake data at 0.25 and 1.25 min in SHH or PHH following the drug-free pre-incubation for varying lengths of time (15, 30, 60, 120, or 180 min). In SHH or PHH that were pre-incubated in the drug-free medium for 60 or 180 min, the initial uptake clearances of PTV and RSV were also measured in the presence of rifamycin SV (30 μM). The processing and quantitation of the samples were performed as described for Uptake Study #1.

### LC–MS/MS Analysis

The drug levels were quantified using an ultra-high-performance liquid chromatography system (Nextera, NY, USA) coupled with a mass spectrometer (Model 8050, Shimadzu, Kyoto, Japan) using the procedure reported previously (8). Briefly, samples (5 μL) were separated using a Kinetex C18 column (2.6 μm, 3 mm × 100 mm; Phenomenex, CA, USA) with the following mobile phase run in gradient mode: 5% B for 1.0 min, 80% B for 4.0 min, 100% B for 1 min, and 5% B for 2 min, where (A) was 0.1% formic acid in water and (B) was 0.1% formic acid in acetonitrile. The total flow rate and the column temperature were set at 0.4 mL/min and 40 °C, respectively. The mass-to-charge (m/z) ratios of the precursor (Q1) and product (Q3) ions were as follows: for CRV, 460 and 356.2; for PTV, 422 and 290.15; for RSV, 482 and 258.1; for PRV, 423 and 321.35; for DHP, 421.3 and 303.25; and for SC-62807, 410.1 and 366.2.

### Model Fitting and Simulation of Uptake Clearance

**Fig. 1:**
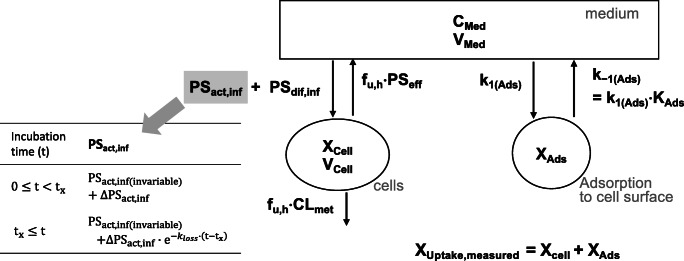
Structure of the kinetic model that describes the drug uptake in isolated hepatocytes. Model equations are provided in the “MATERIALS AND METHODS” section. *C*_Med_, drug concentration in medium; *X*_Cell_, *X*_Ads_, *X*_Uptake_, drug amounts inside cells, drug amount adsorbed to the cell surface, and the apparent amount of drug uptake measured experimentally (the sum of *X*_Cell_ and *X*_Ads_), respectively; *V*_Med_, *V*_Cell_, the respective volumes of medium and cells, respectively; PS_act,inf_, PS_dif,inf_, PS_eff_, CL_met_, the clearance of active uptake, passive influx, passive efflux, and metabolism, respectively; *f*_u,h_, the fraction of unbound drug inside hepatocytes; *k*_1(Ads)_, *k*_-1(Ads)_, the rate constant of adsorption and dissociation, respectively; *K*_Ads_, the equilibrium constant of adsorption; *t* for the incubation time; *t*_x_ for the threshold time up to which PS_act,inf_ is maintained constant; PS_act,inf(invariable)_ and ΔPS_act,inf_ for the active uptake clearance components that are invariable and variable over time, respectively; *k*_loss_ for the rate constant describing the decline in ΔPS_act,inf_ clearance after *t*_x_

Figure 1 shows a kinetic model describing the drug uptake in SHH. The model included three compartments corresponding to cells, medium, and adsorption to the cell surface. Drug adsorption to the cell surface was kinetically defined by the rate constant of adsorption (*k*_1(Ads)_) and dissociation (*k*_–1(Ads)_, defined as the product of *k*_1(Ads)_ and *K*_Ads_ which is an equilibrium constant of adsorption). The constructed model included the following differential equations:

$$ {\displaystyle \begin{array}{c}\frac{\mathrm{d}}{\mathrm{d}\mathrm{t}}{X}_{\mathrm{Cell}}=\left({\mathrm{PS}}_{\mathrm{act},\operatorname{inf}}+{\mathrm{PS}}_{\mathrm{d}\mathrm{if},\operatorname{inf}}\right)\bullet {C}_{\mathrm{Med}}-\left({f}_{\mathrm{u},\mathrm{h}}\bullet {\mathrm{PS}}_{\mathrm{eff}}+{f}_{\mathrm{u},\mathrm{h}}\bullet {\mathrm{CL}}_{\mathrm{met}}\right)\bullet \frac{X_{\mathrm{Cell}}}{V_{\mathrm{Cell}}}\\ {}\frac{\mathrm{d}}{\mathrm{d}\mathrm{t}}{C}_{\mathrm{Med}}\bullet {V}_{\mathrm{Med}}=\left\{-\left({\mathrm{PS}}_{\mathrm{act},\operatorname{inf}}+{\mathrm{PS}}_{\mathrm{d}\mathrm{if},\operatorname{inf}}\right)\bullet {C}_{\mathrm{Med}}+{f}_{\mathrm{u},\mathrm{h}}\bullet {\mathrm{PS}}_{\mathrm{eff}}\bullet \frac{X_{\mathrm{Cell}}}{V_{\mathrm{Cell}}}-{k}_{1\left(\mathrm{Ads}\right)}\bullet {C}_{\mathrm{Med}}\bullet {V}_{\mathrm{Med}}+{k}_{-1\left(\mathrm{Ads}\right)}\bullet {X}_{\mathrm{Ads}}\right\}\\ {}\begin{array}{c}\frac{\mathrm{d}}{\mathrm{d}\mathrm{t}}{X}_{\mathrm{Ads}}={k}_{1\left(\mathrm{Ads}\right)}\bullet {C}_{\mathrm{Med}}\bullet {V}_{\mathrm{Med}}-{k}_{-1\left(\mathrm{Ads}\right)}\bullet {X}_{\mathrm{Ads}}\\ {}{X}_{\mathrm{Uptake},\mathrm{measured}}={X}_{\mathrm{Cell}}+{X}_{\mathrm{Ads}}\\ {}{k}_{-1\left(\mathrm{Ads}\right)}={k}_{1\left(\mathrm{Ads}\right)}\bullet {K}_{\mathrm{Ads}}\end{array}\end{array}} $$

(*X*_Cell,_ *C*_Med_, and *X*_Ads_ for the drug amount inside cells, drug concentration in the medium, and the drug amount adsorbed to the cell surface, respectively; *X*_Uptake,measured_ for the drug amount experimentally measured to be taken up by cells including the amount adsorbed to the cell surface (a sum of *X*_Cell,_ and *X*_Ads_); *V*_Cell_ and *V*_Med_ for the volume of cells and medium, respectively; PS_act,inf_ and PS_dif,inf_ for the uptake clearance via active influx and passive diffusion, respectively; *f*_u,h_, the fraction of unbound drug inside cells; PS_eff_ and CL_met_ for the efflux and metabolic clearance of the total drug, respectively; additional description available in Table II)

We assumed that a decline in the uptake clearance was attributable to a decrease in the active influx (PS_act,inf_). This assumption was based on our experimental results that the uptake clearance of PTV and RSV in the presence of rifamycin SV (30 μM) displayed no declining trend with prolonged pre-incubation in drug-free media (Uptake Study #2; Table I). To model a decline in PS_act,inf_ over time, the following discontinuous functions were used by assuming that up to a certain threshold time (t_x_), PS_act,inf_ is constant [as the sum of the two components that are invariable and variable over time; (PS_act,inf(invariable)_ + ΔPS_act,inf_)] and that beyond *t*_x_, the temporally variable component of ΔPS_act,inf_ declines over time:

$$ {\displaystyle \begin{array}{c}\mathrm{when}\kern0.75em 0\le t<{t}_{\mathrm{x}},\kern0.75em {\mathrm{PS}}_{\mathrm{act},\operatorname{inf}}={\mathrm{PS}}_{\mathrm{act},\operatorname{inf}\left(\mathrm{invariable}\right)}+{\Delta  \mathrm{PS}}_{\mathrm{act},\operatorname{inf}}\\ {}\mathrm{when}\kern1em {t}_{\mathrm{x}}\le t,\kern1.75em {\mathrm{PS}}_{\mathrm{act},\operatorname{inf}}={\mathrm{PS}}_{\mathrm{act},\operatorname{inf}\left(\mathrm{invariable}\right)}+{\Delta  \mathrm{PS}}_{\mathrm{act},\operatorname{inf}}\times {e}^{-{\mathrm{k}}_{loss}\bullet \left(t-{t}_{\mathrm{x}}\right)}\end{array}} $$

**Fig. 2:**
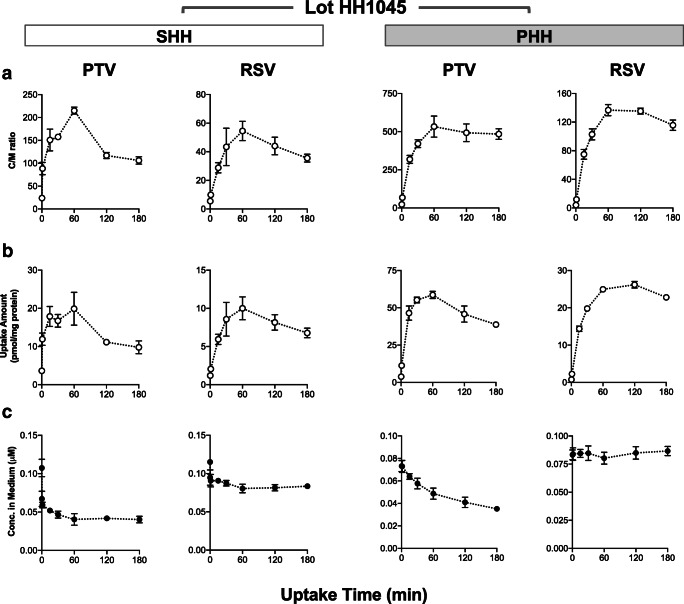
Time courses for the uptake of PTV and RSV in SHH and PHH (lot HH1045) (Uptake Study #1). SHH and PHH (lot HH1045) were exposed to the cocktail dosing solution containing CRV (0.1 μM), PTV (0.1 μM), RSV (0.1 μM), PRV (2 μM), SC-62807 (0.2 μM), and DHP (2 μM) for 0.25, 1.25, 15, 30, 60, 120 or 180 min. For each time point, cell-to-medium (C/M) concentration ratios (**a**), the drug amount taken up by hepatocytes (**b**), and the drug concentrations in the medium (**c**) (mean ± S.D., *n* = 3)

**Table I:** Initial uptake clearances of PTV and RSV in SHH and PHH after varying lengths of drug-free incubation (lot HH1045). The results are shown as mean ± S.D. (*n* = 3)

	PTV: uptake clearance (μL/min/mg protein)				RSV: uptake clearance (μL/min/mg protein)			
Drug-free pre-incubation Time (min)	SHH		PHH		SHH		PHH	
	Rifamycin SV (30 μM)		Rifamycin SV (30 μM)		Rifamycin SV (30 μM)		Rifamycin SV (30 μM)	
	–	+	–	+	–	+	–	+
0	74.0 ± 22.3	31.1 ± 18.1 (by 58%)	117.2 ± 10.6	41.7 ± 13.7 (by 64%)	7.7 ± 5.2	1.7 ± 1.3 (by 78%)	17.0 ± 6.2	4.4 ± 1.4 (by 74%)
15	70.2 ± 7.7		101.6 ± 12.7		7.7 ± 3.0		15.3 ± 4.3	
30	60.9 ± 16.0		108.3 ± 3.3		6.8 ± 3.5		20.4 ± 4.3	
60	59.6 ± 34.3	37.4 ± 11.8 (by 37%)	N.A.	37.2 ± 9.4 (by 65%)^#^	5.0 ± 2.7	2.5 ± 2.2 (by 51%)	N.A.	5.4 ± 0.6 (by 70%)^#^
120	53.2 ± 17.5		111.5 ± 26.8		5.0 ± 2.2		18.7 ± 4.5	
180	58.8 ± 18.7	32.3 ± 16.9 (by 45%)	91.1 ± 5.4	38.8 ± 8.0 (by 57%)	4.2 ± 2.4	1.6 ± 1.6 (by 61%)	18.1 ± 2.3	4.3 ± 1.1 (by 76%)

The numbers shown in the parentheses represent the percentage of the decrease in the uptake clearance values measured in the presence of rifamycin SV (30 μM) compared to the values measured in the absence of rifamycin SV at the corresponding times unless indicated otherwise

^*#*^The uptake clearance value in the absence of rifamycin SV was unavailable at the corresponding time. The percentage of the decrease was thus calculated relative to the average of all the uptake clearance values measured with varying lengths of drug-free incubation (for PTV, 105.9 μL/min/mg protein; for RSV, 17.9 μL/min/mg protein)

(*t* for the incubation time; *t*_x_ for the time threshold up to which PS_act,inf_ is constant; PS_act,inf(invariable)_ and ΔPS_act,inf_ for the active uptake clearance components that are invariable and variable over time, respectively; *k*_loss_ for the rate constant describing the decline in ΔPS_act,inf_ after t_x_)

PS_act,inf(invariable)_ and ΔPS_act,inf_ were optimized by fitting to the observed time course for the uptake of PTV and RSV in SHH (Uptake Study #1; Fig. 2). The *t*_x_ and *k*_loss_ values were estimated from the uptake data of PTV and RSV after varying lengths of drug-free pre-incubation (Uptake Study #2; Table I). The rest of the parameters were fixed as described in Table II.

**Fig. 3:**
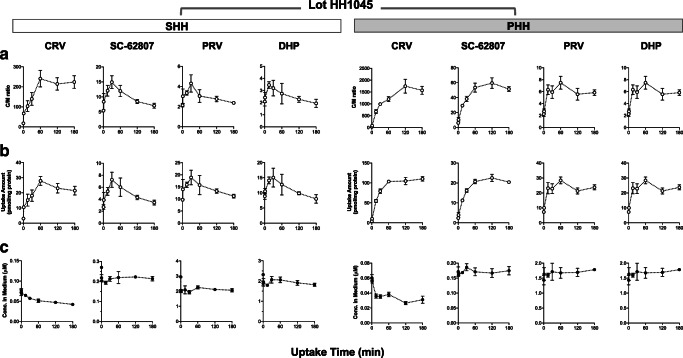
Time courses for the uptake of CRV, SC-62807, PRV, and DHP in SHH and PHH (lot HH1045) (Uptake Study #1). SHH and PHH (lot HH1045) were exposed to the same cocktail dosing solution as described in Fig. 2. For each time point, cell-to-medium (C/M) concentration ratios (**a**), the drug amount taken up by hepatocytes (**b**), and the drug concentrations in the medium (**c**) (mean ± S.D., *n* = 3)

**Fig. 4:**
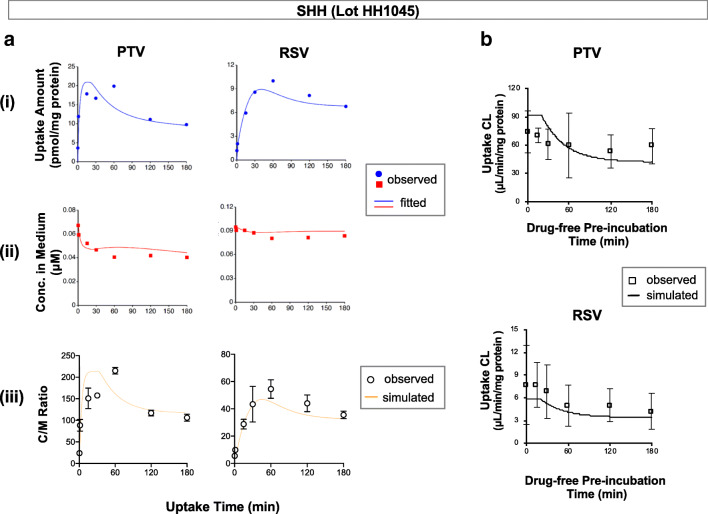
Kinetic modeling of the uptake of PTV and RSV in SHH (lot HH1045). **a** Fitted results are shown in the panels (i) and (ii). The symbols represent the average values of the observed data, and the lines represent the fitted results using the kinetic model. Using the optimized parameters, simulations were performed for the time course of C/M ratios (panel (iii); the symbols represent the observed C/M ratios from Uptake Study #1; the lines represent the simulation results). **b** Using the optimized parameters, simulations were performed for the time courses of the initial uptake clearances for PTV and RSV following varying lengths of drug-free pre-incubation (Uptake Study #2, the symbols represent the observed values summarized in Table I; the lines represent the simulation results)

**Table II:** Summary of the fixed parameters for the hepatocyte model that was fitted to the time course data of the uptake for PTV and RSV in SHH (lot HH1045)

Parameter (unit)	Values used		Description
	PTV	RSV	
*V*_Med_ (μL/mg protein)	1000		The actual volume of medium was normalized to mg protein in cells.
*V*_Cell_ (μL/mg protein)	2.28		From the previous report (7)
*C*_Med(t = 0)_ (μM)	0.0692	0.0957	Extrapolated from the drug concentrations in medium at the two earliest time points (0.25 and 1.25 min; Uptake Study #1)
*X*_Ads(t = 0)_ (pmol/mg protein)	1.55	0.981	Extrapolated from the uptake amount at the two earliest time points (0.25 and 1.25 min;; Uptake Study #1)
*K*_Ads_	44.7	97.6	Estimated by (*C*_Med(t = 0)_·*V*_Med_)/*X*_Ads(t = 0)_ (Uptake Study #1)
*k*_-1(Ads)_ (/min)	10		Chosen based on the assumption of the rapid equilibrium; sensitivity analysis performed to ensure that the selected value is reasonable
PS_dif,inf_ (μL/min/mg protein)	15.6	0.822	Chosen as a half of the uptake clearance in the presence of rifamycin SV (30 μM; based on the projection that rifamycin SV (30 μM) likely inhibited the uptake by OATP1Bs to a partial extent; Uptake Study #2)
*t*_x_ (min)	20		Chosen based on the visual inspection of the threshold time in drug-free pre-incubation, beyond which a decline in the uptake clearance was observed (Uptake Study #2; sensitivity analysis performed by varying the values from 20 to 180 min)
*k*_loss_ (/min)	0.03		Chosen based on the slope of the decline in the uptake clearance (Uptake Study #2)
*f*_u,h_·CL_met_ (μL/min/mg protein)	0.0176	0.0005	For the lot HH1045, there was a decline in the total amount recovered (a sum of the drug amounts detected in cells and medium). The assumption was made that the observed decline is from the metabolic loss. The *f*_u,h_·CL_met_ value was thus calculated as follows: the rate of the presumed metabolic loss (the slope of the decline in the total drug amount recovered from 60 to 180 min) divided by the intracellular drug concentration at 120 min (at the midpoint between 60 and 180 min).
*f*_u,h_·PS_eff_ (μL/min/mg protein)	0.484	0.118	The assumption was made that the steady-state was reached at 180 min with the uptake clearance corresponding to < PS_act,inf_ remaining after a partial loss + PS_dif,inf_ > (estimated from the uptake clearance values at 180 min from Uptake Study #2). By assuming the steady-state (i.e., $\frac{\mathrm{d}}{\mathrm{d}\mathrm{t}}{X}_{\mathrm{Cell}}$=0), the following equation was rearranged to calculate *f*_u,h_·PS_eff_: $\frac{\mathrm{d}}{\mathrm{d}\mathrm{t}}{X}_{\mathrm{Cell}}=\left({\mathrm{PS}}_{\mathrm{act},\operatorname{inf}}+{\mathrm{PS}}_{\mathrm{d}\mathrm{if},\operatorname{inf}}\right)\bullet {C}_{\mathrm{Med}}-\left({f}_{\mathrm{u},\mathrm{h}}\bullet {\mathrm{PS}}_{\mathrm{eff}}+{f}_{\mathrm{u},\mathrm{h}}\bullet {\mathrm{CL}}_{\mathrm{met}}\right)\bullet \frac{X_{\mathrm{Cell}}}{V_{\mathrm{Cell}}}$ At the steady-state, ${f}_{\mathrm{u},\mathrm{h}}\bullet {\mathrm{PS}}_{\mathrm{eff}}=\frac{\left({\mathrm{PS}}_{\mathrm{act},\operatorname{inf}}+{\mathrm{PS}}_{\mathrm{dif},\operatorname{inf}}\right)}{C_{\mathrm{cell}}/{C}_{\mathrm{Med}}}-{f}_{\mathrm{u},\mathrm{h}}\bullet {\mathrm{CL}}_{\mathrm{met}}$

The fitting and simulation analyses were performed using a nonlinear least-squares fitting software, Napp version 2.31 (14), and the weighted sum of squared residual (wSSR) as an objective function for optimization:

$$ \mathrm{wSSR}=\sum \limits_{\mathrm{i}}{\left(\frac{{\mathrm{y}}_{\mathrm{obs},\mathrm{i}}-{y}_{\mathrm{pred},\mathrm{i}}}{{\mathrm{y}}_{\mathrm{obs},\mathrm{i}}}\right)}^2 $$

where *y*_obs, i_ and *y*_pred, i_ are the *i*th observed and *i*th predicted values, respectively.

Using the optimized parameter values, we simulated the time course for the C/M ratios of PTV and RSV (Uptake Study #1) and those for the initial uptake clearances (a sum of PS_act,inf_ and PS_dif,inf_) of PTV and RSV after varying lengths of drug-free pre-incubation (Uptake Study #2).

## RESULTS

### Time Courses for the Drug Uptake in SHH and PHH (Uptake Study #1)

The time courses for the drug uptake in SHH are shown in Figs. 2 and 3 (lot HH1045) and Figs. S1 and S2 (lot HH1052). For lot HH1045, all drugs except CRV displayed the C/M ratios that initially increased and peaked around 30–60 min, followed by a decline (an overshoot phenomenon) (Figs. 2a and 3a). The maximum values of the C/M ratios ranked as follows: CRV > PTV > RSV > SC-62807 > PRV ≅ DHP. For lots HH1052, all drugs except CRV displayed similar overshoot phenomena in their C/M ratios with the peaking times around 15–30 min. The time courses for the drug uptake amount also displayed the overshoot patterns, with the peaking times coinciding with those for the C/M ratios (Figs. 2b and 3b, S1B, and S2B). The drug concentrations in the medium showed minimal changes over time (Figs. 2c and 3c, S1C, and S2C).

The time courses for the drug uptake in PHH are shown in Figs. 2 and 3 (lot HH1045) and Figs. S1 and S2 (lot HH1052). The C/M ratios of all drugs showed an initial increase, but relatively steady over time with no overshoot pattern. The maximum values of the C/M ratios showed a ranking order, similar to those in SHH: CRV > PTV > RSV > SC-62807 > PRV ≅ DHP. The drug uptake amount over time also showed an initial increase and remained mostly steady over time, except PTV showing a slight decline (Figs. 2b and S1B). The drug concentrations in the medium showed minimal changes except CRV and PTV which gradually declined over time.

### Effect of Drug-Free Pre-Incubation on the Uptake of PTV and RSV in SHH and PHH (Uptake Study #2)

In SHH (lot HH1045), the initial uptake clearances of PTV and RSV displayed a partial loss as the drug-free pre-incubation time increased (Table I): the extent of the decrease at 180 min of drug-free pre-incubation compared to the control (no pre-incubation) was approximately 20% (from 74.0 ± 22.3 to 58.8 ± 18.7 μL/min/mg protein) for PTV and 45% (from 7.7 ± 5.2 to 4.2 ± 2.4 μL/min/mg protein) for RSV. In PHH, the initial uptake clearances of PTV and RSV displayed the following changes as the drug-free pre-incubation time increased: the change at 180 min of drug-free pre-incubation was a decrease by 22% (from 117.2 ± 10.6 to 91.1 ± 5.4 μL/min/mg protein) for PTV and an increase by 6% (from 17.0 ± 1.3 to 18.1 ± 2.3 μL/min/mg protein) for RSV.

As expected, rifamycin SV (30 μM) decreased the uptake clearances of PTV and RSV in both SHH and PHH, but their measured values showed no appreciable loss as the drug-free pre-incubation time increased (Table I and Fig. S3). In SHH of lot HH1045 (Table I), the extent by which rifamycin SV inhibited the uptake clearances was 37–58% for PTV and 51–78% for RSV. In PHH, the extent by which rifamycin SV inhibited the uptake clearances was 57–65% for PTV and 70–76% for RSV.

### Kinetic Modeling of the Time Course Data for the Uptake of PTV and RSV in SHH

The hepatocyte model defined PS_act,inf_ as a sum of the components that are invariable and variable over time (PS_act,inf(invariable)_ and ΔPS_act,inf_, respectively) as a discontinuous function (Fig. 1). By fitting to the time course uptake data of PTV and RSV in SHH (Uptake study #1), the values for PS_act,inf(invariable)_ and ΔPS_act,inf_ were optimized as listed in Table III. The extent of a loss in PS_act,inf_ over the 180-min uptake experiment [i.e., the ratio of ΔPS_act,inf_ to PS_act,inf_ (= PS_act,inf(invariable)_ + ΔPS_act,inf_)] was estimated to be 65.3% for PTV and 49.1% for RSV (Table III).

**Table III:** The optimized parameters of PS_act,inf(invariable)_ and ΔPS_act,inf_ (the active uptake clearance components that are temporally invariable and variable, respectively) in the hepatocyte kinetic model

Parameter	Lot HH1045	
	PTV	RSV
PS_act,inf(invariable)_ (μL/min/mg protein)	26.4 ± 8.7	2.6 ± 0.3
ΔPS_act,inf_ (μL/min/mg protein)	49.7 ± 13.9	2.5 ± 0.5
ΔPS_act,inf_/(ΔPS_act,inf_ + PS_act,inf(invariable)_) (%, the percentage of a maximal loss in PS_act,inf_ during the 180-min uptake experiment)	65.3%	49.1%

These two parameters were optimized by fitting the hepatocyte model to the time course data of the uptake amount and concentrations in medium of PTV and RSV (Uptake Study #1; shown in the panels of **a** and **b** of Fig. 2)

The simulated time courses of the C/M ratios using the optimized parameters were in good agreement with the observed data for both PTV and RSV [Figs. 4a(iii)]. Likewise, the simulated profiles of the initial uptake clearances of PTV and RSV in SHH following varying lengths of drug-free pre-incubation (Uptake Study #2) were overall in good agreement with the observed data (Figs. 4b).

## DISCUSSION

Hepatic uptake clearance is well-recognized as one of the important contributors to the overall hepatic handling of anionic drugs, often mathematically explained using the extended clearance concept (1). For anionic drug candidates, quantitative assessment of the hepatic uptake clearance is essential in the prediction of their in vivo pharmacokinetic profiles. Previously, we observed that the C/M ratios obtained in SHH sometimes display an apparent overshoot instead of a steady pattern over time (8). Such an overshoot in the uptake data is problematic in deciding which C/M ratio values (minimum, maximum, or average) to use in estimating the parameters such as the K_p,uu_ at the steady-state (1,8). In contrast to SHH, which have long been used in assessing the hepatic drug uptake, PHH have gained use only in recent years (15–17). Here, we aimed to cross-evaluate the performances of SHH and PHH in assessing the drug uptake of several OATP1B substrates and to identify possible sources for data variability in the uptake experiments thereby enabling informed selection of appropriate experimental systems.

The current study compared the time courses of the drug uptake between SHH and PHH derived from the same lots of human hepatocytes to minimize the inter-batch and inter-donor differences among human primary hepatocytes. The overshoot phenomenon was observed in SHH, but not in PHH (Figs. 2 and 3 for lot HH1045; Figs. S1 and S2 for lot HH1052), supporting that the different culturing conditions may contribute in part to the data variability. The overshoot phenomenon in the C/M ratios was not observed for CRV (Figs. 3 and S2). For CRV, the contribution of the transporter-mediated uptake may be smaller than other statins. Several reports have previously indicated that OATP1Bs might internalize constitutively, and the internalization rates may be accelerated by cellular signaling (e.g., PKC) (11,12). Moreover, OATP1B1 and OATP1B3 appear internalized in cryopreserved human hepatocytes fixed 1 h post-plating, in contrast to human liver sections, which showed that OATP1B1 and OATP1B3 localized predominantly on the membrane surface (18). Based on those findings, we hypothesized that OATP1Bs may internalize from the cell surface over time, decreasing the uptake clearance mediated by OATP1Bs in SHH. We chose two compounds, PTV and RSV (based on their high uptake clearances mainly mediated by OATP1Bs), and assessed their initial uptake clearances in SHH following pre-incubation in the drug-free medium. As shown in Table I, the initial uptake clearances of PTV and RSV decreased with increasing drug-free pre-incubation time. However, the remaining uptake clearances of PTV and RSV in the presence of rifamycin SV (30 μM) showed no declining trend (Table I). Of note, the recent studies reported that the complete inhibition of active influx transporters (both OATP1Bs and non-OATP1B transporters) in hepatocytes requires the rifamycin SV concentrations of 1 mM or higher (15,19). Thus, the measured uptake clearances of PTV and RSV in the presence of rifamycin SV (30 μM) likely include the uptake clearance via active influx by non-OATP1B transporters as well as passive diffusion. By performing the kinetic modeling, we were able to quantify the extent of the active uptake clearance susceptible to a decline when SHH were incubated for an extended time (20 min or longer): 65% for PTV and 49% for RSV (Table III).

The exact mechanisms for a partial, time-delayed loss in the active uptake clearance in SHH warrant further investigations. Using trypan blue exclusion testing, we found no major decline in qualitative cell viability during some of our experiments using SHH (data not shown). We cannot entirely exclude the possible contribution of compromised cell viability to the observed loss in the active uptake clearance. Another possibility that can account for a partial, time-delayed loss in the uptake clearance would be a decline in the driving force for the active uptake. To date, the driving force for OATP1Bs remains unclear: early studies reported the possible involvement of bicarbonate and reduced glutathione as the driving force (20–23), but such findings were not replicated (24). Thus, it is difficult to verify whether a decline in the driving force indeed contributes to the observed decline in the active uptake clearance in SHH. Another potential mechanism may involve the internalization of the transporters from the membrane surface, resulting in a decreased level of functional transporters. It has been reported that PKC activation triggers rapid internalization of OATPs from the membrane. For instance, the treatment with phorbol 12-myristate 13-acetate (a PKC activator) initiated a rapid and reversible internalization of OATP1B1, OATP1B3, or OATP2B1 within 15–60 min (11,12,25). No information is currently available for the internalization rates of OATP1Bs. Further investigations would be necessary to assess the cellular localization status of OATP1Bs in SHH upon prolonged incubation by performing immunolocalization studies (with the treatment of a PKC activator/inhibitor), elucidate the mechanism(s) underlying the observed, time-delayed decline in the uptake clearance in SHH and to assess the internalization rates of OATPs under various culturing conditions. For the compounds used in the current study (particularly, RSV and PTV whose uptake is mediated mainly by OATP1Bs; greater than 70%, Fig. S3), the internalization of OATP1Bs is suspected as a possible mechanism. However, we cannot exclude the possibility for the internalization of other uptake transporters such as OATP2B1 and NTCP, or the upregulation of biliary excretion transporters in SHH with a prolonged incubation time.

In the present study, the C/M ratios and initial uptake clearances for the five tested compounds were higher in PHH than in SHH (Figs. 2 and 3, S1, and S2) Currently, it remains unknown whether PHH and SHH differ in the transporter expression levels. In recent years, targeted proteomics have been increasingly applied to quantify the transporter expression levels in human hepatocytes or liver specimens, but the resulting quantitative data displayed considerable variations, in part from the methodological heterogeneity (e.g., inter-laboratory variabilities including different sample processing methods, inter-lot differences and culturing conditions of hepatocytes). Recently, Kumar *et al.* (26) quantified the levels of the major drug transporters in suspended, plated, and sandwich-cultured human hepatocytes derived from the same donors (*n* = 4). While the expression levels of OATP1Bs were relatively comparable between suspended and plated human hepatocytes derived from the same donor, the inter-individual variability was considerable. In the case of efflux transporters, their expression levels were comparable between suspended and plated human hepatocytes derived from the same donors, but much elevated in SCHH even from the same donors. Considering the data variability reported by Kumar *et al.* (26) and other reports (27), the results may well be preliminary unless the expression levels of OATP1Bs are assessed using a sufficiently large number of hepatocyte lots. As part of ongoing investigations in our laboratories, quantitative targeted proteomics have been applied to measure the major drug transporters in multiple lots of cryopreserved hepatocytes (*n* = 7–10, prepared as PHH or SHH). Upon completion of our analysis, the resulting quantitative data will be compared between PHH and SHH and interpreted in conjunction with the previous results that assessed the transporter expression levels and the associated variability in multiple lots of human hepatocytes (26,28).

Our results indicated that PHH might be less prone to loss of the uptake ability upon prolonged incubation than SHH. Our current finding may also have relevance in the assessment of the *K*_p,uu_ values (commonly used to predict the unbound drug concentrations inside cells that contain the metabolizing enzymes, efflux transporters, and other molecular targets (9)). Yoshikado *et al.* proposed a method that calculates the *K*_p,uu_ values using the steady-state C/M ratios at 37 °C and on ice (8). To ensure that the obtained C/M ratio represents the value at the steady-state (thereby, accurately predicting *K*_p,uu_), it is important to understand the time courses of the C/M ratios and possible factors influencing the time-dependent changes in the uptake measurements. The current study mainly compared the time courses in the OATP1B-mediated uptake measurements between PHH and SHH. SCHH are certainly necessary to evaluate both sinusoidal uptake and biliary excretion clearances of drugs in vitro. For the measurement of the sinusoidal uptake clearance, the use of PHH or SHH is however common, in part by being less labor- and time-intensive than SCHH. The expression of biliary excretion transporters tends to be elevated in SCHH, to the level higher than the liver tissues from the same donors (26). Considering these noted differences from the literature and the current study, the use of PHH is recommended as a convenient tool to assess the sinusoidal drug uptake while maintaining the transporter functions, particularly for OATP1Bs. Depending on the substrates (and the involvement of other transporters, especially, on the canalicular membrane), the use of SCHH may be better suited for the *K*_p,uu_ determination.

## CONCLUSION

The present study indicated that the C/M ratio overshoot observed in SHH might be attributable to a partial loss in the active uptake clearance after a threshold time. PHH are less prone to such changes and therefore are more appropriate for experiments where a prolonged incubation is required, such as estimation of *K*_p,uu_ at the steady-state.

## Electronic supplementary material

ESM 1 **Fig. S1. Time-courses for the uptake of PTV and RSV in SHH and PHH (lot HH1052) (Uptake study #1).** SHH and PHH (lot HH1052) were exposed to the same cocktail dosing solution as described in Fig. 2. For each timepoint, cell-to-medium (C/M) concentration ratios (**A**), the drug amount taken up by hepatocytes (**B**), and the drug concentrations in the medium (**C**) (mean ± S.D., *n* = 3). **Fig. S2. Time-courses for the uptake of CRV, SC-62807, PRV, and DHP in SHH and PHH (lot HH1052) (Uptake study #1).** SHH and PHH (lot HH1052) were exposed to the same cocktail dosing solution as described in Fig. 2. For each timepoint, cell-to-medium (C/M) concentration ratios (**A**), the drug amount taken up by hepatocytes (**B**), and the drug concentrations in the medium (**C**) (mean ± S.D., n = 3). The results for SC-62807 are not available due to inadvertent loss of samples. **Fig. S3. Impact of varying lengths of drug-free pre-incubation on the initial uptake clearance of PTV and RSV in PHH (lot HH1052) (Uptake study #2).** PHH (lot HH1052) were prepared in the same manner as described for Uptake study #1, except that cells were incubated in drug-free media for 0, 15, 30, 60, 120, or 180 min before the measurement of the initial drug uptake clearance (by measuring the drug uptake at 0.25 and 1.25 min). Following varying lengths of drug-free pre-incubation, cells were treated with the cocktail dosing solution as described in Fig. 2. For no drug-free pre-incubation, and drug-free pre-incubation times of 60 and 180 min, the drug uptake was also measured in the presence of rifamycin SV (RifSV, 30 μM). Data are shown as Mean ± S.D. (n = 3). Statistically significant differences among groups were evaluated using the Tukey-HSD (honestly significant difference) test (R version 3.5.0, multicomp package). *, *p* < 0.05; **, *p* < 0.001 (vs. the results obtained in the absence of RifSV at the corresponding time). (PDF 212 kb)

ESM 2 (DOCX 44 kb)

## Abbreviations

SHH: Suspended human hepatocytes
PHH: Plated human hepatocytes
SCHH: Sandwich-cultured human hepatocytes
OATP: Organic anion transporting polypeptide
C/M ratio: Cell-to-medium concentration ratio
K_p,uu_: Unbound hepatocyte-to-medium concentration ratio
PKC: Protein kinase C
PTV: Pitavastatin
RSV: Rosuvastatin
CRV: Cerivastatin
PRV: Pravastatin
DHP: Dehydropravastatin
