# Supplementary figures and images for: Cell-to-Medium Concentration Ratio Overshoot in the Uptake of Statins by Human Hepatocytes in Suspension, but Not in Monolayer: Kinetic Analysis Suggesting a Partial Loss of Functional OATP1Bs
Source: AAPS J. 2020 Oct 15;22(6):133. doi: 10.1208/s12248-020-00512-6 (PMC7561564; doi:10.1208/s12248-020-00512-6)

**Figure S1**

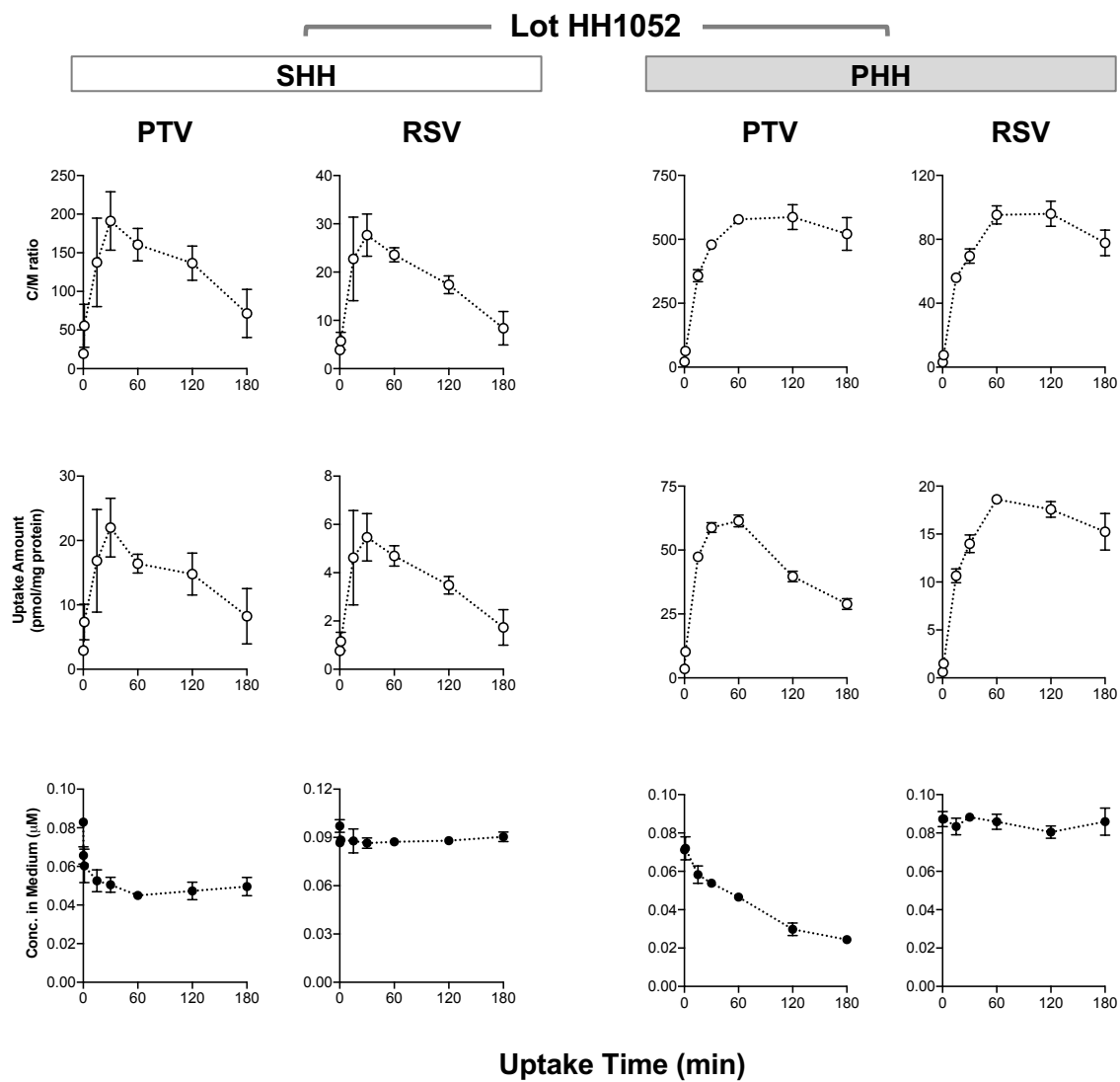

Figure S2

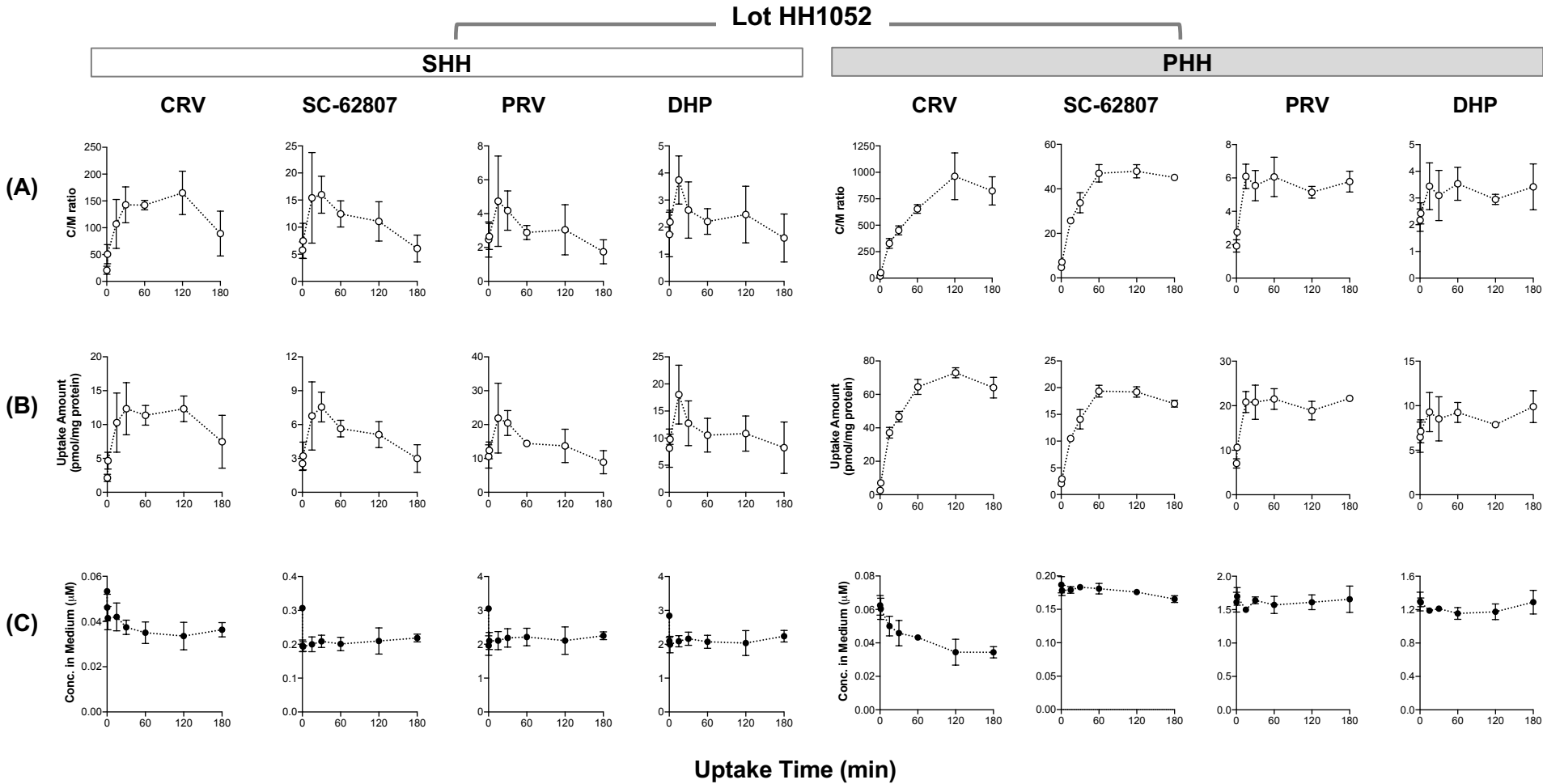

Figure S3

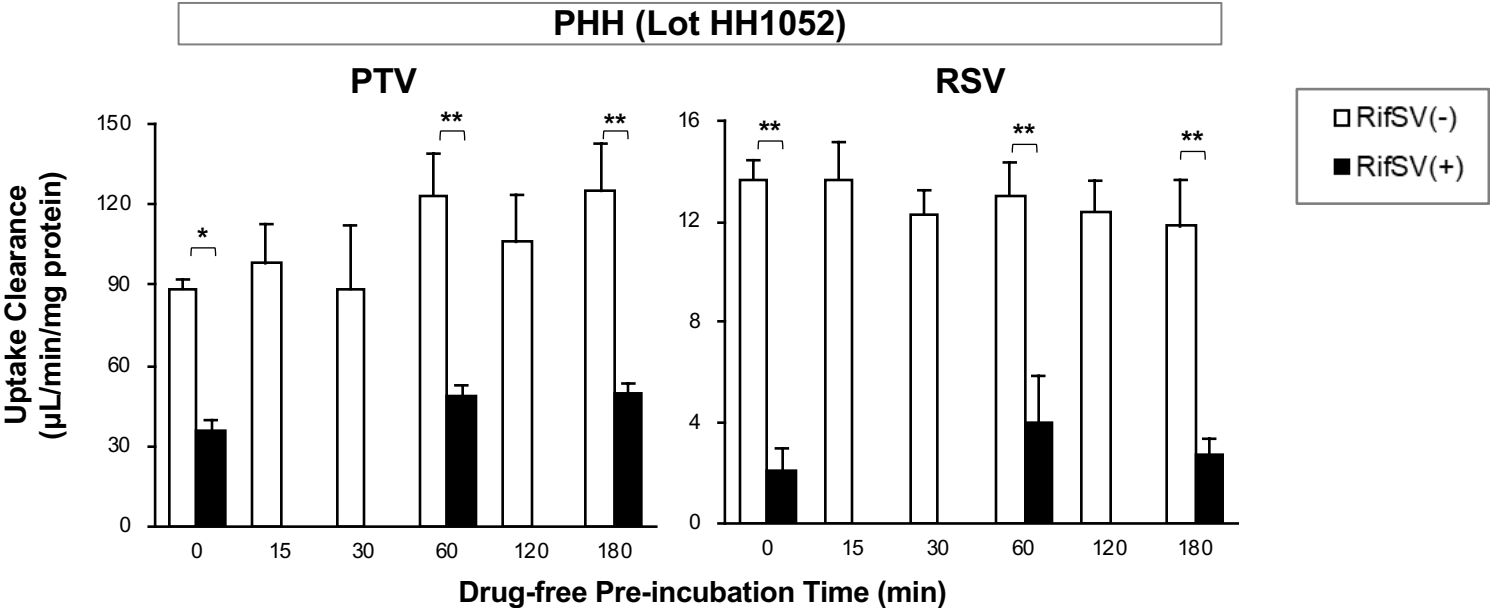

Supplement: Supplementary file 1 — Fig. S1. Time-courses for the uptake of PTV and RSV in SHH and PHH (lot HH1052) (Uptake study #1). SHH and PHH (lot HH1052) were exposed to the same cocktail dosing solution as described in Fig. 2. For each timepoint, cell-to-medium (C/M) concentration ratios (A), the drug amount taken up by hepatocytes (B), and the drug concentrations in the medium (C) (mean ± S.D., n = 3). Fig. S2. Time-courses for the uptake of CRV, SC-62807, PRV, and DHP in SHH and PHH (lot HH1052) (Uptake study #1). SHH and PHH (lot HH1052) were exposed to the same cocktail dosing solution as described in Fig. 2. For each timepoint, cell-to-medium (C/M) concentration ratios (A), the drug amount taken up by hepatocytes (B), and the drug concentrations in the medium (C) (mean ± S.D., n = 3). The results for SC-62807 are not available due to inadvertent loss of samples. Fig. S3. Impact of varying lengths of drug-free pre-incubation on the initial uptake clearance of PTV and RSV in PHH (lot HH1052) (Uptake study #2). PHH (lot HH1052) were prepared in the same manner as described for Uptake study #1, except that cells were incubated in drug-free media for 0, 15, 30, 60, 120, or 180 min before the measurement of the initial drug uptake clearance (by measuring the drug uptake at 0.25 and 1.25 min). Following varying lengths of drug-free pre-incubation, cells were treated with the cocktail dosing solution as described in Fig. 2. For no drug-free pre-incubation, and drug-free pre-incubation times of 60 and 180 min, the drug uptake was also measured in the presence of rifamycin SV (RifSV, 30 μM). Data are shown as Mean ± S.D. (n = 3). Statistically significant differences among groups were evaluated using the Tukey-HSD (honestly significant difference) test (R version 3.5.0, multicomp package). *, p < 0.05; **, p < 0.001 (vs. the results obtained in the absence of RifSV at the corresponding time). (PDF 212 kb) [file 12248_2020_512_MOESM1_ESM.pdf]
