# Supplementary material for: Cell-to-Medium Concentration Ratio Overshoot in the Uptake of Statins by Human Hepatocytes in Suspension, but Not in Monolayer: Kinetic Analysis Suggesting a Partial Loss of Functional OATP1Bs
Source: AAPS J. 2020 Oct 15;22(6):133. doi: 10.1208/s12248-020-00512-6 (PMC7561564; doi:10.1208/s12248-020-00512-6)
Supplement: Supplementary file 2 — (DOCX 44 kb) [file 12248_2020_512_MOESM2_ESM.docx]

Supplementary table

**Table S1**. Kinetic parameters for the uptake of drugs in human primary hepatocytes or *in vitro* cell line models

| compound | Model used | **K_m_**  (μM) | Ref |
| --- | --- | --- | --- |
| cerivastatin | Primary human hepatocytes  (from 3 different lots) | 18.3 ± 6.9  2.6 ± 1.5  3.7 ± 1.3 | (1) |
|  |  |  |  |
|  |  |  |  |
| pitavastatin | Primary human hepatocytes | 11.5 ± 2.2 | (2) |
|  | Primary human hepatocytes | 3.0 ± 0.8 | (3) |
|  | HEK293/OATP1B1 | 2.5 ± 0.1 | (4) |
|  | HEK293/OATP1B1 | 1.3 ± 0.3 | (5) |
|  | HEK293/OATP1B1 | 3.0 ± 0.4 | (6) |
|  | HEK293/OATP1B3 | 3.3 ± 0.4 | (6) |
|  | HEK293/OATP1B1 | 4.8 ± 0.7 | (7) |
|  |  |  |  |
| rosuvastatin | Primary human hepatocytes (plated) | 4.7 ± 0.8 | (8) |
|  | Primary human hepatocytes | 10.3 ± 1.9 | (9) |
|  | HEK293/OATP1B1 | 15.3 ± 3.0 | (9) |
|  | HEK293/OATP1B3 | 13.5 ± 2.0 | (9) |
|  | HEK293/OATP1B1 | 9.3 | (4) |
|  | HEK293/OATP1B1 | 0.8 ± 0.2 | (10) |
|  | HEK293/OATP1B3 | 14.2 ± 2.8 | (10) |
|  | HEK293/OATP1B1 | 9.0 ± 0.1 | (5) |
|  | HEK293/OATP1B1 | 13.1 ± 0.4  12 ± 6.3 | (11) |
|  |  |  |  |
| pravastatin | Primary human hepatocytes in the presence of human plasma (plated) | 76.8 ± 27.4 | (12) |
|  | HEK293/OATP1B1 | 29.0 ± 2.7 | (5) |
|  | HEK293/OATP1B1 | 27.0 ± 8.1 | (4) |
|  |  |  |  |
| dehydropravastatin | HEK293/OATP1B1 | 6.5 ± 2.2 | (14) |
|  | HEK293/OATP1B3 | 17.1 ± 8.3 | (14) |
|  |  |  |  |
| SC-62807 | HEK293/OATP1B1 | 260 ± 138 | (13) |
|  | HEK293/OATP1B3 | 19.8 ± 2.6 | (13) |

**References**

1. Shitara Y, Itoh T, Sato H, Li AP, Sugiyama Y. Inhibition of transporter-mediated hepatic uptake as a mechanism for drug-drug interaction between cerivastatin and cyclosporin A. J Pharmacol Exp Ther. 2003;304(2):610-6. doi: 10.1124/jpet.102.041921.

2. Nakai D, Nakagomi R, Furuta Y, Tokui T, Abe T, Ikeda T, et al. Human liver-specific organic anion transporter, LST-1, mediates uptake of pravastatin by human hepatocytes. J Pharmacol Exp Ther. 2001;297(3):861-7.

3. Fujino H, Nakai D, Nakagomi R, Saito M, Tokui T, Kojima J. Metabolic stability and uptake by human hepatocytes of pitavastatin, a new inhibitor of HMG-CoA reductase. Arzneimittelforschung. 2004;54(7):382-8. doi: 10.1055/s-0031-1296988.

4. Izumi S, Nozaki Y, Maeda K, Komori T, Takenaka O, Kusuhara H, et al. Investigation of the impact of substrate selection on in vitro organic anion transporting polypeptide 1B1 inhibition profiles for the prediction of drug-drug interactions. Drug Metabo Dispos 2015;43(2):235-47. doi: 10.1124/dmd.114.059105.

5. Sharma P, Butters CJ, Smith V, Elsby R, Surry D. Prediction of the in vivo OATP1B1-mediated drug-drug interaction potential of an investigational drug against a range of statins. Eur J Pharm Sci. 2012;47(1):244-55. doi: 10.1016/j.ejps.2012.04.003.

6. Hirano M, Maeda K, Shitara Y, Sugiyama Y. Contribution of OATP2 (OATP1B1) and OATP8 (OATP1B3) to the hepatic uptake of pitavastatin in humans. J Pharmacol Exp Ther. 2004;311(1):139-46. doi: 10.1124/jpet.104.068056.

7. Soars MG, Barton P, Ismair M, Jupp R, Riley RJ. The development, characterization, and application of an OATP1B1 inhibition assay in drug discovery. Drug Metabo Dispos 2012;40(8):1641-8. doi: 10.1124/dmd.111.042382.

8. Liao M, Zhu Q, Zhu A, Gemski C, Ma B, Guan E, et al. Comparison of uptake transporter functions in hepatocytes in different species to determine the optimal model for evaluating drug transporter activities in humans. Xenobiotica. 2019;49(7):852-62. doi: 10.1080/00498254.2018.1512017.

9. Shen H, Yang Z, Mintier G, Han YH, Chen C, Balimane P, et al. Cynomolgus monkey as a potential model to assess drug interactions involving hepatic organic anion transporting polypeptides: in vitro, in vivo, and in vitro-to-in vivo extrapolation. J Pharmacol Exp Ther. 2013;344(3):673-85. doi: 10.1124/jpet.112.200691.

10. Kitamura S, Maeda K, Wang Y, Sugiyama Y. Involvement of multiple transporters in the hepatobiliary transport of rosuvastatin. Drug Metabo Dispos 2008;36(10):2014-23. doi: 10.1124/dmd.108.021410.

11. van de Steeg E, Greupink R, Schreurs M, Nooijen IH, Verhoeckx KC, Hanemaaijer R, et al. Drug-drug interactions between rosuvastatin and oral antidiabetic drugs occurring at the level of OATP1B1. Drug Metabo Dispos 2013;41(3):592-601. doi: 10.1124/dmd.112.049023.

12. Mao J, Doshi U, Wright M, Hop C, Li AP, Chen Y. Prediction of the Pharmacokinetics of Pravastatin as an OATP Substrate Using Plateable Human Hepatocytes With Human Plasma Data and PBPK Modeling. CPT Pharmacometrics Syst Pharmacol. 2018;7(4):251-8. doi: 10.1002/psp4.12283.

13. Takashima T, Wu C, Takashima-Hirano M, Katayama Y, Wada Y, Suzuki M, et al. Evaluation of breast cancer resistance protein function in hepatobiliary and renal excretion using PET with 11C-SC-62807. J Nucl Med. 2013;54(2):267-76. doi: 10.2967/jnumed.112.110254.

14. Kaneko K, Tanaka M, Ishii A, Katayama Y, Nakaoka T, Irie S, et al. A Clinical Quantitative Evaluation of Hepatobiliary Transport of [(11)C]Dehydropravastatin in Humans Using Positron Emission Tomography. Drug Metabo Dispos 2018;46(5):719-28. doi: 10.1124/dmd.118.080408.
